# More visual mind wandering occurrence during visual task performance: Modality of the concurrent task affects how the mind wanders

**DOI:** 10.1371/journal.pone.0189667

**Published:** 2017-12-14

**Authors:** HeeSun Choi, Michael Geden, Jing Feng

**Affiliations:** Department of Psychology, North Carolina State University, Raleigh, North Carolina, United States of America; University of British Columbia, CANADA

## Abstract

Mind wandering has been considered as a mental process that is either independent from the concurrent task or regulated like a secondary task. These accounts predict that the form of mind wandering (i.e., images or words) should be either unaffected by or different from the modality form (i.e., visual or auditory) of the concurrent task. Findings from this study challenge these accounts. We measured the rate and the form of mind wandering in three task conditions: fixation, visual 2-back, and auditory 2-back. Contrary to the general expectation, we found that mind wandering was more likely in the same form as the task. This result can be interpreted in light of recent findings on overlapping brain activations during internally- and externally-oriented processes. Our result highlights the importance to consider the unique interplay between the internal and external mental processes and to measure mind wandering as a multifaceted rather than a unitary construct.

## Introduction

**Fig 1:**
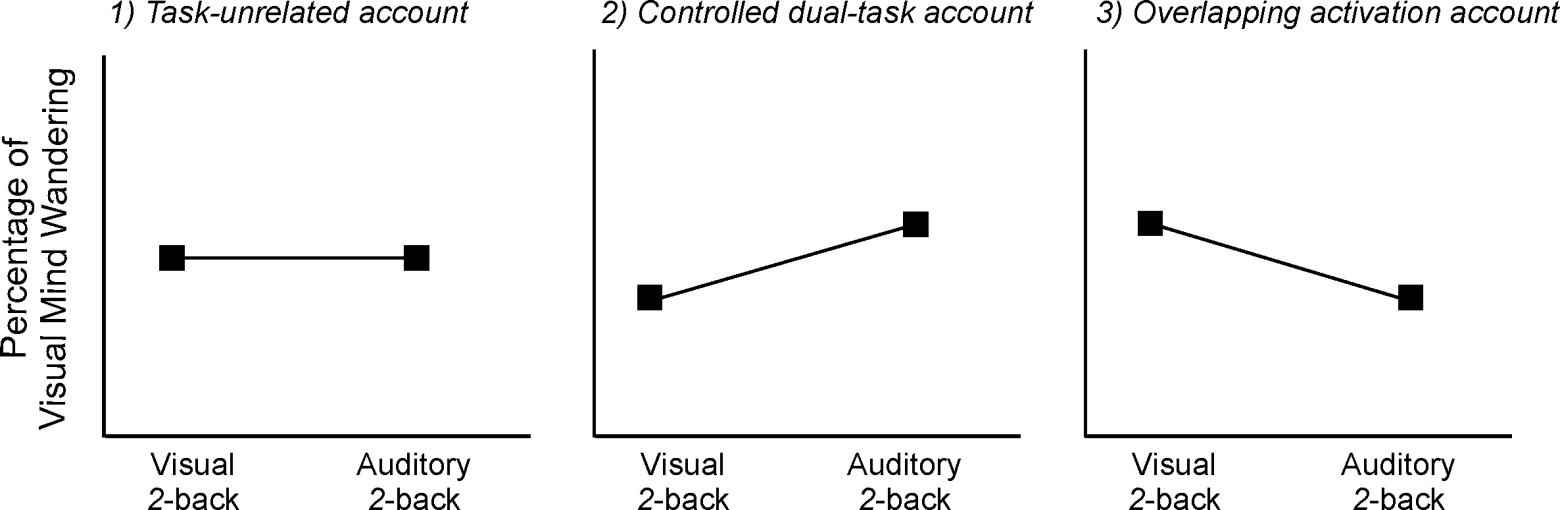
Three possible predicted relations between task modality and form of mind wandering. 1) Mind wandering is task-unrelated, 2) mind wandering is greater in a modality different from the task modality, and 3) mind wandering is greater in the task modality.

Our minds often drift away from the present task and wander around task-unrelated thoughts [1,2]. Recent research suggests that the experience of mind wandering is multi-dimensional and the form of thoughts during mind wandering varies along unique dimensions such as the modality and level of intrusiveness or detail [3,4]. In particular, the modality of the thoughts is a unique dimension in which visual imagery, like a film, and auditory forms, like an audiobook, are in opposition, with particular brain areas linked to each of the two forms [3–5]. Examining whether and how the modality form of mind wandering relates to the form of the concurrent task can provide valuable insights into the nature of mind wandering, as current understandings make mixed predictions (Fig 1).

Mind wandering has been traditionally defined as being independent or unrelated to the concurrent task [6]. It is described as an internal train of thought, separated from the external environment [7]. During mind wandering, attention to the external input is likely to be reduced, and the mind is diverted to and focused on internal task-unrelated thoughts rather than external inputs [8,9]. Because mind wandering is unrelated to the concurrent task and associated with reduced attention to the external input, the content and form of mind wandering is likely to be independent to the concurrent task. This *task-unrelated account* may predict that the modality form of mind wandering would not be affected by the form of the concurrent task.

On the other hand, certain evidence suggests that the concurrent task does have an impact on mind wandering, as the rate of mind wandering depends on the demand of the task [10,11]. The more demanding the task, the less we mind wander. A perspective which sees that a cognitive system functions in an adaptive manner within a given context to minimize the risk of obstructing concurrent task performance emphasizes cognitive capacity to regulate the occurrence of mind wandering [8,12]. This perspective may suggest that mind wandering would be regulated and inhibited when the continuous cognitive resource is needed for the concurrent task. Such regulation is much like when we juggle two external tasks. Given the visual and auditory modalities possess separate mental resources [13,14], this *controlled dual-task account* predicts that the modality form of mind wandering is regulated to reflect the least competition for a mental resource with the concurrent task. Therefore, mind wandering should tend to be in a different modality form as the task when a person is trying to engage in and perform the task successfully.

A third possible and *newer* account stems from neuroimaging findings that internally-oriented processes (e.g., visual and auditory imageries) and externally-oriented processes (e.g., perception) of the same information involve overlapping brain networks [15–17]. A recent meta-analysis of neuroimaging studies of mind wandering revealed the significant involvement of many brain regions outside the default mode network that could be modality specific [18]. For example, the lingual gyrus, an area for high-level visual processing [19], was speculated to be involved in visual mind wandering [20]. Similarly, the perigenual cingulate cortex and a region of the caudal posterior cingulate cortex were found to be associated with modality-specific mind wandering [5]. It was suggested that stimulating certain brain areas may increase the propensity of mind wandering [21]. According to this *overlapping activation account*, it is possible that a visual task activates brain regions that support both visual perception and imagery, making visual mind wandering to occur more frequently or become more accessible by consciousness. This account suggests that mind wandering is more likely in the same form as the concurrent task.

In this study, we examined the relation between the modality forms of mind wandering and the concurrent task by measuring the frequency of mind wandering in different modality forms during performing visual or auditory external tasks. Given the essential involvement of executive function in mind wandering [22,23], we matched the demand on the central executive function between the visual and auditory tasks by presenting a 2-back task in visual and auditory forms. In addition, we adopted a within-subject design, as individual variations in visual-verbal cognitive style [24] may impact participants’ experienced form of mind wandering. Each participant completed three task conditions: simple fixation task, visual 2-back, and auditory 2-back. Using the probe-caught method, we measured the rate and form of mind wandering, as well as participants’ performance on the 2-back tasks.

## Materials and methods

### Participants

A total of 35 undergraduate students (17 males, 18 females; Age *M* = 19.66, *SD* = 1.66) were recruited from a research university. Participants were students enrolled in an introductory psychology course and received course credits for experiment participation.

### Tasks

#### Fixation task

**Fig 2:**
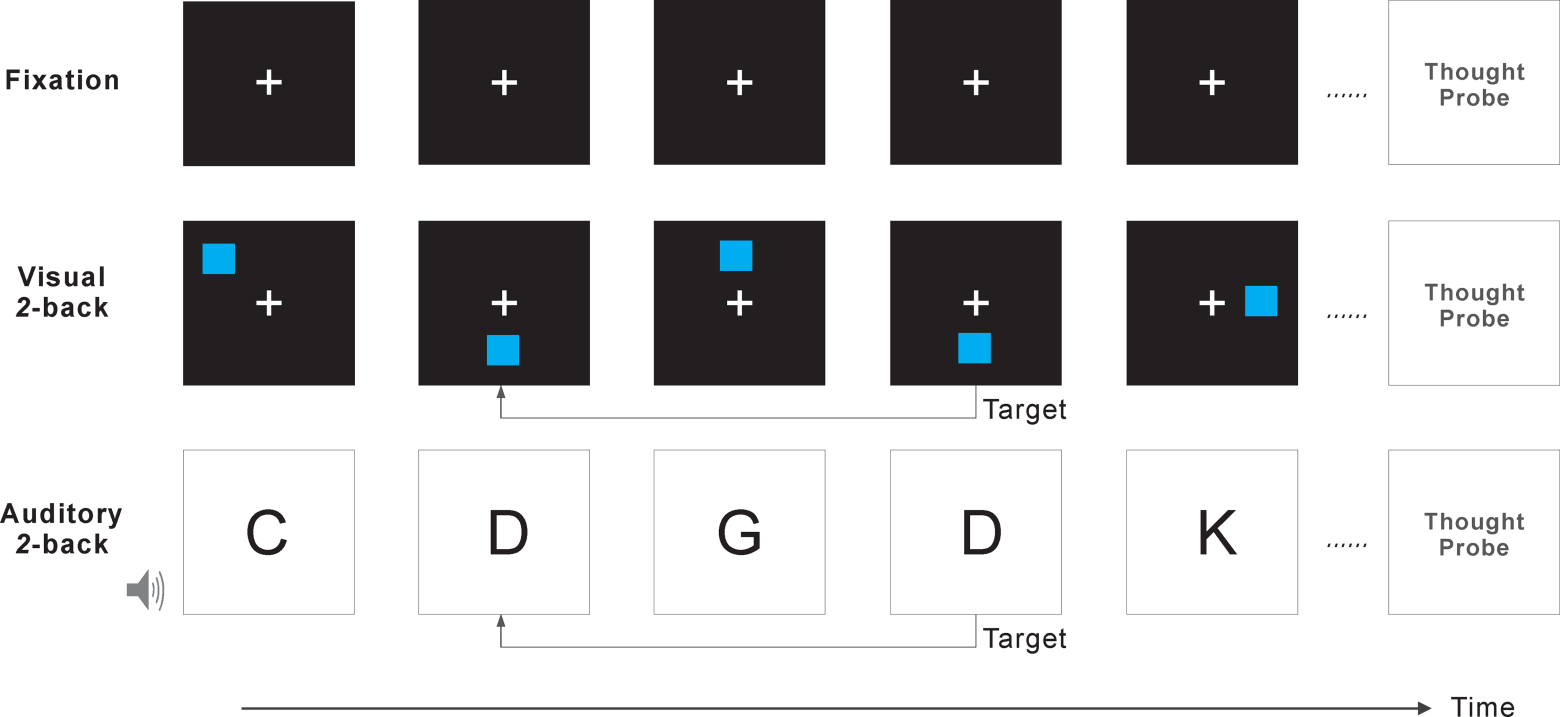
Illustration of the three task conditions. Fixation, visual 2-back, and auditory 2-back.

During a simple fixation task, participants were instructed to fixate on a cross (“+”, Fig 2) displayed in the center of the screen. No response was required. This was used as a baseline condition to assess a participant’s general dominant form of mind wandering when a concurrent task did not demand a particular form of perceptual processing.

#### 2-back tasks

The task is a version of the n-back task which has been widely used to assess working memory [25,26]. We adopted and modified the *2*-back task developed by Jaeggi and Buschkuehl at the Working Memory and Plasticity Laboratory at the University of California, Irvine (download from http://wmp.education.uci.edu/software/). The task was administered using E-Prime (Version 2.0, Psychology Software Tools, Pittsburgh, PA) on a PC. During the visual 2-back task, a sequence of displays each containing one blue square at one of the eight possible locations on the screen (randomly selected from one of the locations in a 3×3 grid centered on the fixation, except the center location) was presented. Participants were asked to monitor the location of each blue square. If the location of a square matched with the location of the square that occurred two displays ago (e.g., bottom middle → upper middle → bottom middle, Fig 2), participants were instructed to press the spacebar. Similarly, in the auditory 2-back task, letters (randomly selected from C, D, G, K, P, Q, T, and V, one letter at a time) were presented through headphones. Participants were instructed to press the space bar if a letter corresponded with the letter before the last (e.g., D → G → D, Fig 2). Participants were asked to maintain the fixation on the cross displayed at the center of the screen during both the visual and auditory tasks. Each visual or auditory stimulus was presented for 500 ms, followed by a 3000 ms interval. The visual and auditory tasks were identical except the modality form of the stimuli.

#### Thought probes

To sample mind wandering, we used the probe-caught method [27,28]. During each task, thought probes appeared at pre-determined quasi-random intervals. A total of 80 thought probes were presented, including 20 probes during the fixation task, 30 probes during the visual 2-back task, and 30 probes during the auditory 2-back task. During the 2-back tasks, probes were presented with an average interval of 15 trials (range from 13 to 17 trials). The varying interval was used to minimize participants’ anticipation. Because each 2-back task trial lasted 3500 ms, the average interval between thought probes was 52.5 seconds. The probe intervals were matched to be consistent in the fixation task. However, because no task performance was measured in the fixation task and it only aimed to assess baseline mind wandering propensity, a shorter task time was used. In the current study, mind wandering was defined as the internal thought that is unrelated to a concurrent task, without explicitly distinguishing the dependency on external inputs (See Stawarczyk et al. [29] for classification of stimulus-dependency and task-relatedness). Thus, each probe asked what a participant was thinking about just prior to the probe: thinking “about the task” (i.e., on-task), or thinking “about something else” (i.e., mind wandering on task-unrelated thoughts). Participants were instructed to report that they were thinking “about the task,” if they’ve been focused on the task. Thus, during the n-back tasks, thinking about the stimuli and the response would be “about the task”. Similarly, during the fixation task, thinking about maintaining the fixation is regarded as being on-task. During the instruction, participants were also given examples of task-unrelated thoughts such as thinking about recent or impending events, thinking about current conditions (e.g., hunger or sleepiness), daydreams, and fantasies disconnected from reality [30], that were not related to the current task. If a participant indicated that he/she was on-task, the 2-back task resumed with no further inquiry. If the participant reported thinking “about something else”, he/she was further asked to indicate whether the thoughts were in the form of “images (like a television program or film)” or “words (like an inner monologue or audiobook)” [3]. There was one additional question asking about the temporal dimensions of the thoughts (i.e., “in the past,” “in the future,” or “in the here and now, or with no specific time” derived from Jackson, Weinstein & Balota [31]). Because the temporal dimensions of mind wandering is out of the scope of this paper, results are not included here.

#### Procedures

The experimental procedures were approved by the North Carolina State University Institutional Review Board. Each participant was first given a brief introduction of the experiment and signed a consent form. Participants were instructed that they would be asked to indicate their state of mind (focusing on the task or mind wandering) during the tasks. They were asked to maintain a focus on the task, but they were also told that it would be natural to mind-wander periodically. Thus, they should honestly report their state of mind. Each participant completed both the visual and auditory 2-back tasks as well as the fixation task. The order of three task conditions was counterbalanced across participants, and instruction was provided before each task. A short practice was also given before each of the auditory and visual tasks. The fixation task took about 20 minutes, and each of the visual and the auditory 2-back tasks took approximately 30 minutes. Participants were given short rests between tasks.

## Results

To validate the non-differential demands of our visual and auditory tasks, we first compared participants’ accuracy on the 2-back tasks between the visual and auditory tasks. A repeated measures ANOVA indicated that participants showed comparable accuracy on the visual and auditory tasks (visual 2-back task: *M* = .73, *SE* = .04, 95% CI = [.66, .80], False Alarm Rate = .05; auditory 2-back task: *M* = .74, *SE* = .03, 95% CI = [.68, .82], False Alarm Rate = .03), *F*(1,34) = .29, *p* > .25, η_*p*_^2^ = .08, suggesting that the two tasks did not differ in difficulty.

### Rates of mind wandering across task conditions

The rate of mind wandering was calculated using the number of mind wandering reports divided by the total number of thought probes in each task condition. Across the three task conditions, participants mind wandered 47% of the time (*M* = .47, *SE* = .04, 95% CI = [.39, .54]). We conducted a repeated measures ANOVA to compare the rate of mind wandering across the three task conditions (i.e., fixation, visual 2-back, and auditory 2-back). Mauchly’s test indicated the assumption of sphericity had not been violated (χ^2^(2) = 4.26, *p* = .12) thus no correction was made. Participants mind wandered more during the fixation task (*M* = .68, *SE* = .05, 95% CI = [.59, .78]) than during the 2-back task conditions (visual *2*-back: *M* = .38, *SE* = .05, 95% CI = [.28, .47]; auditory *2*-back: *M* = .34, *SE* = .04, 95% CI = [.25, .42]), *F*(2,68) = 44.17, *p* < .001, η_*p*_^2^ = .57. This result suggested that participants experienced more mind wandering when they were in the fixation task than when performing the 2-back tasks.

### Modality forms of mind wandering across task conditions

**Fig 3:**
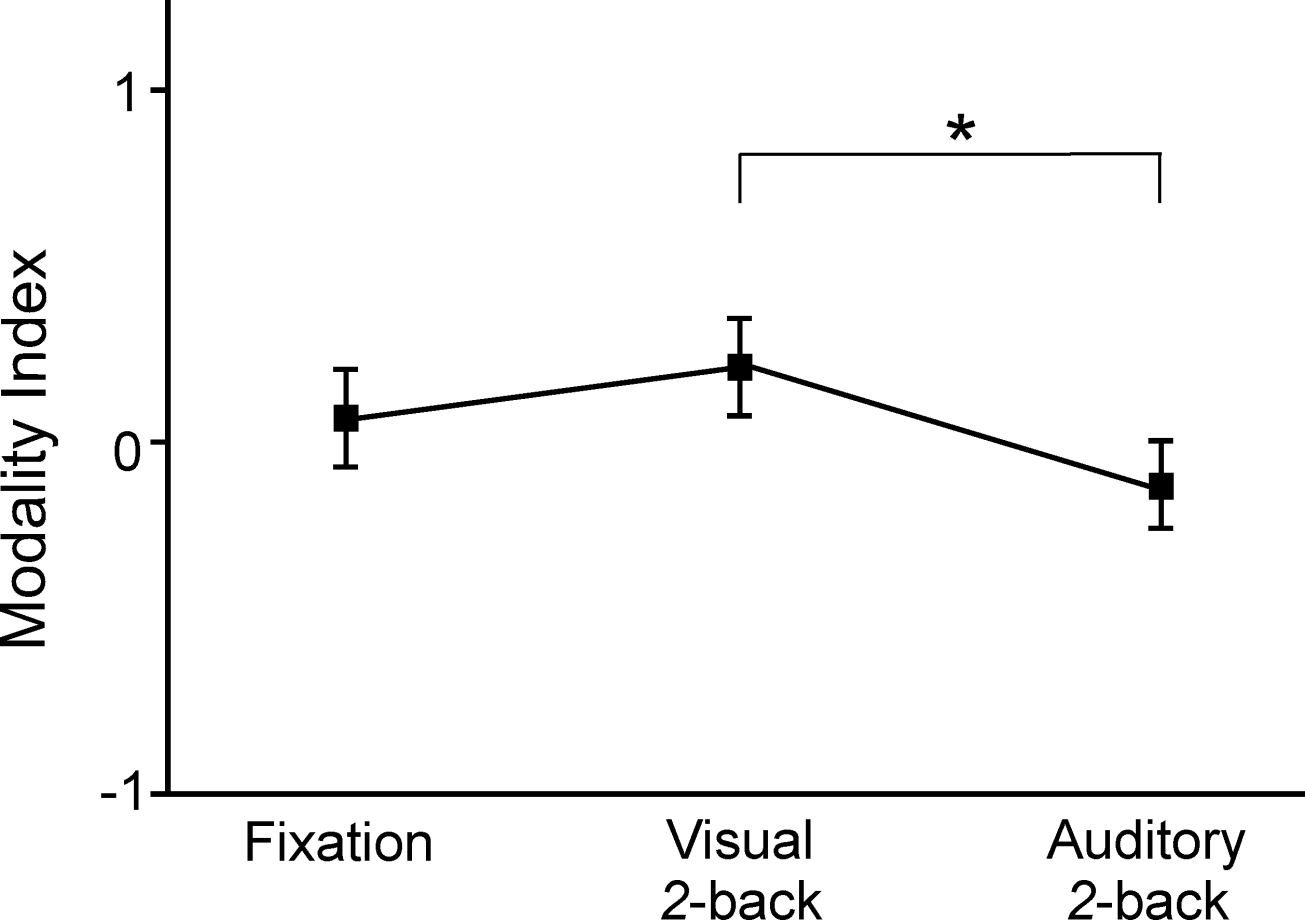
Observed relations between task modality and form of mind wandering. Modality index of -1 indicating mind wandering being always auditory and 1 being always visual. Error bars represent ±1SE. Observation indicated mind wandering is greater in the same modality as the concurrent task.

We computed an index of the modality of mind wandering from the differences between visual and auditory mind wandering reports divided by all mind wandering reports during each task performance (i.e., [the number of visual mind wandering reports–the number of auditory mind wandering reports] / the total number of mind wandering reports). The modality index ranged -1 to 1 with -1 indicating mind wandering being always auditory and 1 being always visual. If the index approaches 0, it would indicate the frequencies of visual and auditory forms of mind wandering are comparable. Preliminary analysis indicated that there were large individual differences in the modality form of mind wandering (fixation condition: *M* = .08, *SD* = .68, ranged -1.00 to 1.00; visual condition: *M* = .22, *SD* = .68, ranged -1.00 to 1.00; auditory condition: *M* = -.15, *SD* = .61, ranged -1.00 to .89). Using a repeated measures ANOVA, we compared the mind wandering modality among the tasks of fixation, visual 2-back, and auditory 2-back (Fig 3). Two participants were excluded in this analysis because they did not report any mind wandering during at least one of the task conditions. Therefore, data from 33 participants were analyzed. Mauchly’s test indicated that the assumption of sphericity had not been violated (χ^2^(2) = 4.65, *p* = .10) thus no correction was made. The ANOVA results indicated significant differences in mind wandering modality among the three tasks, *F*(2,64) = 5.31, *p* = .007, η_*p*_^2^ = .14. When comparing between the visual and auditory tasks, participants were more likely to experience the form of mind wandering that was consistent with the concurrent task’s perceptual modality: more visual forms of mind wandering occurred when performing the visual task (*M* = .22, *SE* = .12, 95% CI = [-.02, .46]; 61% visual and 39% auditory mind wandering) and more auditory forms of mind wandering occurred during the auditory task (*M* = -.10, *SE* = .10, 95% CI = [-.31, .11]; 45% visual and 55% auditory mind wandering), and the modality index score was significantly different between the two 2-back tasks, *p* = .002. Pairwise comparisons also indicated that the modality index was significantly lower (i.e., mind wandering occurred more in auditory forms) in the auditory task condition compared to the fixation condition (*M* = .08, *SE* = .12, 95% CI = [-.15, .31]; 54% visual and 46% auditory mind wandering), *p* = .04. Although it was not statistically significant (*p* = .24), a consistent trend was found for the visual task condition showing increased occurrences of visual forms of mind wandering compared to the fixation task condition. These results suggested that a concurrent external task facilitates the propensity of mind wandering in the same modality.

### Effects of mind wandering on task performance

We examined the effect of mind wandering on task performance, by comparing task accuracy across the three mind states: on-task, intra-modal mind wandering (i.e., auditory mind wandering during auditory task or visual mind wandering during visual task), and cross-modal mind wandering (i.e., auditory mind wandering during visual task or visual mind wandering during auditory task). Task accuracy for each mind state was calculated using the trials before each report of the mind state. For example, if a participant reported the mind being on-task after a probe, task trials from immediately after the response to the previous probe to just before the response (e.g., “on-task”) to the current probe were classified as on-task trials. In the case of dual-tasking with two external tasks, intra-modal tasks (i.e., two external tasks presented in the same modality) interfered much greatly than cross-modal tasks (i.e., two external tasks presented in different modalities) (e.g., [32]). The purpose of this analysis was to examine whether the interference of mind wandering follows the same pattern as the interference of an external secondary task on a primary task. Due to the variable nature of self-reported mind wandering (e.g., no report on the visual form of mind wandering during the auditory task), this analysis only included data from 18 participants who exhibited all combinations of mind states by task forms. We conducted a 3 (mind state: on task, intra-modal mind wandering, cross-modal mind wandering) × 2 (task: visual 2-back, auditory 2-back) repeated measures ANOVA. Mauchly’s test indicated the assumption of sphericity had not been violated for either the main effect of mind state (χ^2^(2) = 1.83, *p* = .40) or the interaction (χ^2^(2) = 3.86, *p* = .15) thus no correction was made. Task accuracy differed significantly among the three mind states, *F*(2,34) = 7.26, *p* = .002, η_*p*_^2^ = .30, with a significant interaction between mind state and task form, *F*(2,34) = 5.51, *p* = .008, η_*p*_^2^ = .25. We also conducted a *planned contrast* with Bonferroni correction comparing accuracies between the intra-modal and cross-modal mind wandering states, on each of the visual and auditory tasks. During the visual task, there was no accuracy difference between mind wandering in the same form of the concurrent task (i.e. intra-modal, *M* = .67, *SE* = .06, 95% CI = [.55, .79]) and in a different form (i.e., cross-modal, *M* = .62, *SE* = .07, 95% CI = [.47, .76]), *p >* .*25*. During the auditory task, there was a significant difference that participants were more accurate when mind wandering in a different form as the concurrent task (i.e., cross-modal, *M* = .81, *SE* = .05, 95% CI = [.70, .92]) than when wandering in the same form (i.e., intra-modal, *M* = .69, *SE* = .05, 95% CI = [.58, .79]), *p =* .*03*. This suggested that in the case of mind wandering, the difference between intra-modal and cross-modal interferences was not as clear as the case of two external tasks. However, it is important to note that, this exploratory analysis only included a small sample size (n = 18), and the time window of trials being associated with a report of a mind state is larger than typical (to accommodate the sparse occurrence of targets in 2-back tasks), thus the comparison on interferences on task performance between intra-modal and cross-modal mind wandering was preliminary and further examination is needed.

To explore performance cost within a shorter time window, we conducted further analysis associating 3 trials prior to each probe (an interval that lasted approximately 10.5 seconds). This is a smaller time window which is within the typical range used in the literature. One important issue to note is that such analysis is largely based on target-absent trials (thus correct rejection and false alarms, rather than hits and misses), therefore the interpretation of results also requires much caution. In this further analysis, task accuracy was modelled using a generalized estimation equation for a binomial family dependent variable with the canonical logit link function. Generalized estimation equations were used due to their flexibility in the distribution of the response variable and correlation structure which can account for within-subject correlation [33]. A random effect was fit for each participant and trial. Task accuracy differed significantly among the three mind states, χ^2^(2) = 59.1, *p* < .001, with a significant interaction between mind state and task form, χ^2^(2) = 9.87, *p* = .007. During the visual task, there was a significant difference between mind wandering in the same form of the concurrent task (i.e. intra-modal, M = .86, 95% CI = [.83, .88]) and in a different form (i.e., cross-modal, M = .90, 95% CI = [.87, .92]), χ^2^(1) = 5.55, *p* = .019. During the auditory task, there was a significant difference in that participants were more accurate when mind wandering in a different form as the concurrent task (i.e., cross-modal, M = .93, 95% CI = [.90, .95]) than when wandering in the same form (i.e., intra-modal, M = .89, 95% CI = [.86, .91]), χ^2^(1) = 9.58, *p* = .002.

## Discussion

Contrary to the predictions by the accounts that mind wandering is independent of the concurrent task or is regulated as a secondary task (i.e., the form of mind wandering is unaffected by or different from that of the concurrent task), our results suggest that mind wandering is more likely to occur in the same form as the concurrent task, which would support the *overlapping activation account*. This result implies an intriguing and unique interplay between internal and external mental processes. Findings from imaging research further support this account by showing a significant overlap on brain regions recruited for the internal and external mental processes of information in the same form (e.g., visual perception and visual imaginary) [15,17,34]. Processing perceptual information may have increased the propensity of mind wandering in the same form. Alternatively, stimulating certain brain areas by external inputs may possibly increase the propensity or awareness of internal processing of mind wandering [18,21]. In addition, compared to the auditory stimuli and the mere fixation, the visual stimuli in the current experiment may have promoted more eye movements by participants. Given the strong connection between mental imagery and eye movements [35,36], additional oculomotor feedback during the visual 2-back task may have increased activation and stimulated more visual imagery. Examining this speculation requires monitoring participants’ eye movements during the tasks in a future study. Furthermore, our results showed that the modality effect on the mind wandering cost was only significant for the auditory task (i.e., participants were more accurate when mind wandering in a different form [visual] as the auditory concurrent task), but not for the visual task. While this finding requires further experimental exploration, the current results do not rule out the possibility that auditory processing differs from visual processing in the sense that it is the form that suffers more from intra-modal mind wandering.

We also found significant individual differences in the modality form of mind wandering. This individual difference may explain the discrepancy in findings between our study and Antrobus et al. [37], which—to our knowledge—is the only other study that has examined the modality forms of mind wandering under different task modality conditions and found the results supporting that task-irrelevant thought is greater in non-task modality. In Antrobus et al. [37], one group of participants performed a visual task, and another group performed an auditory task, and half of each group was asked to report either only visual or auditory mind wandering. Although the findings of this study indicate that with an increasing task demand, the internal imagery was inhibited more strongly in the same sensory modality as the concurrent task than in the different modality, sizable individual variances could have played a significant role in the results from this completely between-subject design. Examining trait-level differences in the form of mind wandering could further enhance our understanding of mind wandering.

There is an increasing awareness that mind wandering is a multidimensional construct [5,8,27,38,39]. Such fundamental shift in the perspective on mind wandering requires us to revisit the conceptualizations that were largely based on unitary measures of mind wandering which may not capture different modalities or types of mind wandering. Our research demonstrates the importance of using emerging measures of the multiple dimensions of mind wandering and individual differences in these dimensions to expand our understanding of mind wandering.
